# Editorial: Novel Imaging Techniques in the Management of Thyroid Nodules and Autoimmune Thyroid Disease

**DOI:** 10.3389/fendo.2019.00804

**Published:** 2019-11-20

**Authors:** Ewelina Szczepanek-Parulska, Joanna Klubo-Gwiezdzinska, Marek Ruchala

**Affiliations:** ^1^Department of Endocrinology, Metabolism and Internal Medicine, Poznan University of Medical Sciences, Poznan, Poland; ^2^Thyroid Tumors and Functional Thyroid Disorders Section, National Institute of Diabetes and Digestive and Kidney Diseases (NIDDK), Bethesda, MD, United States

**Keywords:** thyroid nodules, thyroid cancer, Graves Disease, subacute thyroiditis, autoimmune thyroid disease

The high incidence of thyroid nodules, ranging from about 10 up to 70% of adult population, constitute a major socioeconomic problem (1). Wide access to high-resolution thyroid ultrasonography (US) and other imaging techniques has dramatically raised the detection rate of thyroid lesions (2), but only 8–18% of thyroid nodules in general population are malignant (3). Therefore, an everyday diagnostic challenge for endocrinologists is to identify the patients with higher risk of malignancy, who should undergo prompt diagnostics and surgical management. The gold standard of thyroid nodules diagnostics is conventional ultrasonography followed by fine-needle aspiration biopsy (FNAB) of qualified lesions. However, biopsy is an invasive procedure, which has certain limitations as up to 10–25% patients undergoing biopsy receive inconclusive cytological result (4–6). Hence, there is still a need to search for novel imaging procedures or markers, that would allow a non-invasive estimation of malignancy risk with satisfying sensitivity and specificity. Recent studies have demonstrated that novel imaging techniques might be also used as an additional diagnostic tool to monitor and differentiate different types of thyroiditis (7). The goal of this Research Topic collection is to present current trends and progress that novel techniques of thyroid imaging brings to diagnostics, monitoring and therapy of thyroid nodules and autoimmune thyroid disease (AITD).

In the recent years, the sonoelastography has been introduced as a promising tool to differentiate between benign and malignant thyroid lesions (8, 9). Although current data reveal that diagnostic value of sonoelastography is inferior compared with FNAB, this technique is still considered as a very useful additional method increasing diagnostic accuracy of conventional ultrasound. Consistently, Dobruch-Sobczak et al. in a study including 208 patients with 305 thyroid nodules concludes that decreased elasticity of thyroid nodules is associated with increased risk of malignancy that may justify more aggressive management. In the study by Liu et al. authors employed specific MRI modalities and demonstrated its potential usefulness in differentiation between benign and malignant thyroid nodules. Zonally oblique multi-slice diffusion-weighted imaging (area under curve—AUC = 0.937) proved to be superior to amide proton transfer (AUC = 0.783), *p* = 0.028. Authors conclude that these modalities might constitute a non-invasive, promising method for improving differential diagnosis of thyroid nodules in a clinical setting. Appropriate management of thyroid nodules in pediatric population constitutes a particularly difficult task, i.e., due to possible presence of ectopic intrathyroidal thymic tissue and the similarity of its US pattern to thyroid carcinoma. Sonographic features allowing for initial differential diagnosis of such ectopic tissue and malignant lesions are presented in the study by Stasiak et al. The authors also demonstrated a significant role of sonoelastography in differential diagnosis of such lesions, and presented examples of its application.

Another specific subgroup of patients are subjects diagnosed with low-risk papillary thyroid microcarcinoma. There has been an ongoing debate on the possibility of active surveillance (AS) instead of surgical therapy in these patients. That approach is endorsed by the Japanese Thyroid Society as well as by the American Thyroid Association and European guidelines on the management of thyroid nodules and thyroid cancer (6, 10–13). The comprehensive review article by Xue et al. summarizes the current state of knowledge on the role of imaging techniques implemented in AS and involves clues helpful during follow-up of these patients. Authors indicate limited value of US in detecting extra-thyroid extension or lymph node metastases, which could be increased by combining it with computed tomography (CT) scan. Although patients with AS approach are at slightly higher risk of lymph node metastases than those managed surgically, still the incidence remains low in both groups and disease specific survival and overall survival is similar. Moreover, authors underline the role of genetic biomarkers that might be helpful to differentiate between the low and high-risk thyroid cancers and discuss ethical issues concerning the process of qualification of patients to be surveilled instead of surgically managed.

Due to current wider accessibility of MRI, more and more patients would have an incidentally detected thyroid pathology found by this imaging modality. Hence, the MRI-based image characteristics of thyroid nodules and diffuse thyroid pathologies, such as AITD, need to be appropriately recognized and interpreted. Kang et al. addresses this issue and summarizes incidental thyroid findings in a group of 387 patients with neck MRI performed as a part of diagnostic workup for non-thyroidal illness and present the MRI characteristics of the thyroid images found in patients with evidence of AITD. The main MRI characteristics of AITD consist of high and inhomogeneous signal intensity on T2-weighted images. Another study by Malkowski et al. demonstrated that patients with AITD differ from subjects with normal thyroid also in terms of standardized uptake value (SUV-max) of the thyroid parenchyma measured using 18F-FDG-PET/CT. Given increasing accessibility to 18F-FDG-PET/CT imaging worldwide, it is important to correctly interpret and verify increased diffused radioisotope uptake detected in patients undergoing scan for non-thyroidal reasons. According to American Thyroid Association guidelines, focally increased uptake in the thyroid needs verification by US, and lesions above 10 mm in size require FNAB. However, incidentally detected abnormal thyroid PET scan pattern may also be a first manifestation of AITD and requires further diagnostics toward the presence of AITD.

There is an evidence that Color Doppler examination might be a useful predictive factor for Graves' disease (GD) relapse (14). In addition, there is an ongoing effort to identify novel imaging modalities as well as specific serum biomarkers to assist in identification of patients at highest risk of GD relapse. Identification of such predictors would help in clinical decision making to stratify which patients are the best candidates for conservative management with the highest chance for remission on pharmacotherapy, and which may benefit from definitive therapy (ablation with radioiodine or total/near total thyroidectomy). The study by Struja et al. evaluated potential usefulness of application of a high-throughput proton NMR metabolomic profile in prediction of relapse of the GD. However, only a moderate prognostic potential was demonstrated. Out of 227 studied markers, pyruvate and triglycerides in medium VLDL were selected as candidates with acceptable discriminatory strength as predictors with AUCs of 0.73 and 0.67, respectively.

There is an evidence of specific genotypes predisposing to subacute thyroiditis and AITD. However, certain genetic factors are still to be identified. In the study by Stasiak et al. it has been demonstrated that sonographic pattern of subacute thyroiditis might be associated with the specific HLA-haplotype. The authors reported a significant association between the HLA^*^B18:01 and the US characteristics of subacute thyroiditis. In the study by Jia et al. a novel relationship between CD14 gene polymorphism and AITD has been described. The authors also point out that the allele model, recessive model, and homozygous model of rs2569190 and rs2915863 embodied strong correlations with GD after the adjusting of age and gender. This association was the strongest for female patients and those with a positive family history of GD. Additionally, an analysis of CD14 expression was studied in thyroid tissues derived from thyroidectomized patients with GD, but its role in pathogenesis of GD requires further investigations.

In summary, a constant progress and rising accessibility of modern imaging techniques, as well as identification of novel biochemical and genetic markers gradually improve our understanding of pathogenesis of focal and diffuse thyroid pathologies. Combining standard diagnostic procedures with novel imaging techniques plays more and more important role in the clinical decision making in these patients.

## Author Contributions

All authors listed have made a substantial, direct and intellectual contribution to the work, and approved it for publication.

### Conflict of Interest

The authors declare that the research was conducted in the absence of any commercial or financial relationships that could be construed as a potential conflict of interest.
